# Navigating Psychotrauma: The Role of Perceived Social Support in Coping Strategies Among Young Adults

**DOI:** 10.7759/cureus.72368

**Published:** 2024-10-25

**Authors:** Muddsar Hameed, Habiba Ali, Hamna Atiq, Aleena Waseem, Khadeejah Iqbal, Syeda Hadeeqa Aqeel, Aliyah Usman Qureshi, Syeda Masooma Naqvi, Sheraz Khan Afridi, Ismael Ahmad

**Affiliations:** 1 Department of Neuroscience, Brain Tech Clinic and Research Center, Islamabad, PAK; 2 Department of Psychology, Quaid e Azam University, Islamabad, PAK; 3 Department of Clinical Psychology, Shifa Tameer e Millat University, Islamabad, PAK; 4 Department of Internal Medicine, Fatima Jinnah Medical University, Lahore, PAK; 5 Department of Clinical Psychology, National University of Science and Techonology, Islamabad, PAK; 6 Department of Internal Medicine, Combined Military Hospital (CMH) Lahore Medical College and Institute of Dentistry, Lahore, PAK; 7 Department of Physiotherapy, Rawalpindi Medical University Pakistan, Rawalpindi, PAK; 8 Department of Nursing, Ariana Medical University, Nangarhar, AFG

**Keywords:** coping strategies, gender differences, perceived social support, psycho-trauma, trauma experiences

## Abstract

Introduction

The present study aimed to examine the role of experience of psycho-trauma and perceived social support in the coping strategies of young adults. Moreover, it aimed to determine the role of demographic variables such as gender, age, education of respondents, paternal and maternal education, occupation, income, and nature of trauma experienced across the study variables.

Methods

The study utilized a quantitative cross-sectional survey design to examine the role of psycho-trauma, perceived social support, and coping strategies among 352 young adults aged between 18 and 28 years from Pakistan and Afghanistan (Islamabad and Jalalabad, respectively). Participants were selected through convenience sampling from various public and private universities. Data were collected using standardized instruments, including the Brief Trauma Questionnaire (BTQ), the Multidimensional Scale of Perceived Social Support (MSPSS), and the Brief Coping Orientation to Problems Experienced (COPE) Inventory.

Results

Results showed significant findings regarding the relationships between psycho-trauma, perceived social support, and coping strategies among a sample of 352 young adults. Inferential statistics revealed substantial negative correlations between psycho-trauma and both perceived social support (r = -0.18, p < 0.01) and problem-focused coping (r = -0.21, p < 0.01). Regression analysis indicated that psycho-trauma explained 19% of the variance in coping strategies, while perceived social support accounted for 29%. Gender differences were observed, with men scoring higher on problem-focused coping (t = 4.66, p < 0.02) and women scoring higher on emotion-focused coping (t = 3.12, p < 0.00).

Conclusion

The study also highlighted significant differences based on trauma experiences, education levels, and parental education and occupation, underscoring the complex interplay between these factors and their impact on young adults coping mechanisms. Study implications, limitations, and suggestions for future research have also been discussed.

## Introduction

Psychological trauma arises from distressing events overwhelming an individual's coping mechanisms, such as witnessing death, physical injury, assault, sexual abuse, extreme poverty, or verbal abuse [1]. As per the World Health Organization's (WHO) statistics, injuries account for an estimated 5.8 million fatalities each year. In particular, traumatic incidents stand tall as the primary cause of death prevalent among people aged between five and 44 years [2].

Trauma refers to external physical damage, while psychological trauma is an individual's subjective emotional experience of overwhelming events or perceived threats to life, integrity, or sanity [3]. Developmental trauma, involving adverse childhood experiences like abuse or violence, increases the likelihood of mental health issues like post-traumatic stress disorder (PTSD), characterized by avoidance, negative thinking, and emotional instability [4].

Trauma can stem from events that violate personal beliefs and rights, causing confusion and insecurity. This includes physical threats to survival and security and betrayals by trusted institutions. Only 8% of people develop PTSD, with vulnerability linked to mental and biological predispositions, early childhood trauma, and trauma severity [5].

Perceived social support may be defined as the perception of functions that are to be performed for an individual by significant others, such as family members, friends, and co-workers, who can provide informational, appraisal instrumental, and/or emotional assistance [6]. Perceived social support is a crucial factor in an individual's well-being, particularly during trauma or illness [7]. Theories like social-cognitive, social control, and stress and coping perspectives contribute to understanding perceived social support [8]. The social-cognitive perspective explains the relationship between mental health and perceived social support. This model suggests that positive mental health is directly influenced by social support, which can mitigate negative emotions and thoughts. Negative evaluations and unenthusiastic emotions can cause stress, but the availability of social support reduces the likelihood of negative thoughts and promotes positivity [9].

Coping is defined as the thoughts and behaviors mobilized to manage internal and external stressful situations. It is a term used distinctively for conscious and voluntary mobilization of acts, different from 'defense mechanisms' that are subconscious or unconscious adaptive responses, both of which aim to reduce or tolerate stress [10]. Coping strategies are essential when stressors are perceived as disparate from available resources. They involve active attempts to resolve stress or manage situations. Four main types of coping strategies are identified: active avoidance, problem-focused, positive, and religious/denial [11].

Social support is crucial for traumatic event recovery, with family and friends' support being more effective [12]. Mental health issues worldwide are increasing due to violence, political instability, and societal changes. Understanding psychopathy in Pakistan requires a holistic view, with social support crucial for improving quality of life [13]. In Pakistan, these issues are alarming due to violence and social disruption [14].

This study is significant as it explores the complex interplay between psycho-trauma experiences, perceived social support, and coping strategies among young adults. By examining these relationships, the research provides valuable insights into how students manage and cope with traumatic events, emphasizing the crucial role of social support in enhancing resilience and mental health.

Rationale of the study

Psycho-trauma, causing fear, sadness, guilt, anger, or grief, can lead to mental health issues like PTSD, depression, anxiety, and substance abuse. Gender affects trauma symptoms, with females experiencing higher rates. Trauma perceptions influence coping strategies, with active coping putting survivors in power. Relaxation techniques and social support are essential for managing negative reactions.

Therefore, the article aims to highlight the current persistent violence in Pakistani and Afghani society as a consequence of the war against terrorism, with a particular focus on the District of Swat and the province of Nangarhar and its potential link to psychological trauma. Previous research indicating effective models of healthcare in similar war situations is also examined to provide an evidence base for models to be used in the area. Finally, possible strategies to help provide psychological support are discussed.

Pakistan and Afghanistan have experienced a surge in violence in the past years, causing psychological trauma and helplessness among survivors. Mental health services struggle due to a lack of resources, trained staff, policy realization, and awareness programs. Effective social assistance and culturally compatible interventions are needed.

Objectives of the study 

The objective of this study is to determine the role of experience of psycho-trauma, perceived social support, and coping strategies among young adults and also investigate the role of demographic variables (gender, age, education of respondents, university affiliation, paternal and maternal education, occupation, income, and nature of trauma) in the major construct of the study.

## Materials and methods

Research design

This study employed a quantitative survey method with a cross-sectional design to investigate the experiences of psycho-trauma and perceived social support in coping strategies among young adults.

Sample

A convenience sample of 352 young adults (university students) from Islamabad, Pakistan, and Jalalabad, Afghanistan, including both males (n = 135) and females (n = 217), participated in the study. Participants were affiliated with public and private sector universities. The age range of respondents varied from 18 to 28 years (mean (M) = 23; SD = 2.8). Education levels included undergraduate (n = 150), master's (n = 159), Master of Philosophy (MPhil) (n = 40), and Doctor of Philosophy (PhD) (n = 3). The demographic variables collected were age, gender, education level, paternal and maternal education, occupation, income, and trauma experienced.

Table 1 represents the distribution of the sample based on gender, age, education of respondents, institutional affiliation, paternal and maternal education, occupation, income, and trauma experienced as well.

**Table 1:** Demographic details of the sample (N=352) F: frequency; %: percentage; MPhil: Master of Philosophy; PhD: Doctor of Philosophy

Variables	F	%
Age (years)	-	-
18-22	183	52
23-28	166	47.2
Gender	-	-
Female	217	61.6
Male	135	38.4
Education of respondents	-	-
Graduation	150	42.6
Masters	159	45.2
MPhil	40	11.4
PhD	3	0.9
Institutional affiliation	-	-
Public sector	259	73.6
Private sector	93	26.4
Paternal education	-	-
None	8	2.3
Matriculation	93	26.4
Graduate	74	21
Masters	135	38.4
PhD	36	10.2
Others	6	1.7
Maternal education	-	-
None	99	28.1
Matriculation	131	37.2
Graduate	79	22
Masters	36	10
PhD	1	2
Paternal income (in Pakistani Rupees)	-	-
<50,000	85	24.1
50,000 – 100,000	99	28.1
>100,000	168	47.7
Maternal income (in Pakistani Rupees)	-	-
<50,000	294	83.5
50,000 – 100,000	36	10.2
>100,000	21	6
Paternal occupation	-	-
None	14	4
Business	74	21
Government job	192	54.5
Private job	72	20.5
Maternal occupation	-	-
None	244	69.3
Business	4	1.1
Government job	55	15.6
Private job	48	13.6
Trauma experienced	-	-
None	158	44.9s
Once	94	26.7
Twice	43	12.2
Multiple	57	16.2

Instruments

The Brief Trauma Questionnaire (BTQ), derived from the Short Trauma Interview [15], is a self-reported questionnaire designed to assess exposure to traumatic events based on The Diagnostic and Statistical Manual of Mental Disorders, 5^th^ edition (DSM-5) Criterion A (Appendix A). It focuses on life-threatening or severely injurious experiences. The Multidimensional Scale of Perceived Social Support (MSPSS) developed by Rafai [16], is a 12-item scale that measures perceived social support from family, friends, and significant others (Appendix B). Each dimension is assessed with four items using a five-point Likert scale. Higher scores indicate greater perceived social support. The scale has a reliability coefficient of α = 0.85. The Brief Coping Orientation to Problems Experienced (COPE) Inventory, originally developed by Carver [17] and translated into Urdu by Akthar (2005), is a 28-item self-report measure of coping strategies, rated on a four-point Likert scale [18]. It includes subscales for problem-focused coping, active avoidant coping, religious/denial coping, and emotion-focused coping (Appendix B). Higher scores indicate greater use of the respective coping strategies. A comprehensive demographic sheet was used to collect information on age, gender, education level, university affiliation, paternal and maternal education, occupation, income, and trauma experienced among young adults.

Procedure

Data were collected from young adults using standardized survey instruments between March 2024 and May 2024. The research was approved by the Institutional Review Board (IRB) of Brain Tech Clinic and Research Center, Islamabad, Pakistan (IRB-BTC-10098). Ethical standards were maintained throughout the study. Participants were informed about the study's objectives, and their consent was obtained online before starting the questionnaire. All participants were above 18 years of age; therefore, no parental or guardian consent was required. The ethical committee allowed the use of electronic consent as the study involved only questionnaires and posed no harm to participants. The anonymity and confidentiality of the participants were ensured, and the data were used exclusively for research purposes.

## Results

Alpha reliability coefficients and descriptive statistics

To see the descriptive and psychometric properties of alpha reliability coefficients, mean standard deviation, range, skewness, and kurtosis of the brief trauma questionnaire, perceived social support, brief cope inventory, and their subscales.

Table 2 illustrates the alpha reliability of coefficient values, indicating that the highest reliability was found on coping strategies, i.e., 0.90, followed by its subscales, which were active-avoidant, problem-focused, religious/denial, and emotion-focused coping. The values ranged from 0.77, 0.70, 0.60, and 0.75, and for the BTQ, Cronbach alpha was 0.80. Cronbach’s alpha of perceived social support was 0.88, followed by its subscales family, friends, and significant others; the values ranged from 0.82, 0.79, and 0.82 skewness, and kurtosis -1 to +1 showed that data were normally distributed. Mean and standard deviation were computed to determine the general average scores of participants on particular scales used in this study, whereas the value of skewness showed the distribution of scores among variables for perceived social support, its subscale (family, friends, and significant others), coping strategies, and its subscales (active-avoidant, problem-focused, religious/denial, and emotion-focused coping), and psycho-trauma.

**Table 2:** Descriptive statistics and alpha coefficients of study variables (N = 352) M: mean; SD: standard deviation; α: Cronbach's alpha; Skew: skewness; Kurt: kurtosis; AA: active avoidant; PF: problem-focused; R/D: religious/denial; EF: emotion-focused; FM: family; FS: friends; O: others; Psy.tr: psycho-trauma; PSS: perceived social support; CS: coping strategies

Variables	No. of items	α	M	SD	Skew	Kurt	Range	
							Actual	Potential
Psy. Tr	20	0.80	35.05	3.59	-0.81	1.08	20-40	20-40
CS	28	0.90	71.66	14.22	-1.04	1.74	28-106	28-112
AA	10	0.77	19.77	4.43	-0.9	0.99	7-28	7-28
PF	7	0.70	24.72	5.18	-0.69	0.9	10-37	10-40
R/D	4	0.60	9.45	2.74	-0.14	-0.41	4-16	4-16
EF	7	0.75	17.71	4.14	-1.12	1.34	7-28	7-28
PSS	12	0.88	46.67	9.6	-0.72	0.14	12-60	12-60
FM	4	0.82	16.18	3.75	-0.96	0.35	4-20	4-20
FS	4	0.79	9.26	2.34	-0.41	-0.03	4-16	4-20
O	4	0.82	15.35	4.07	-0.8	-0.12	4-20	4-20

Table 3 displays the correlation matrix for the role of experiencing psycho-trauma and perceived social support in the coping strategies of young adults; moreover, trauma included divorce, natural disaster, accident, and loss of a loved one. Results showed that psycho-trauma was significantly negatively correlated with perceived social support and problem-focused coping strategy, while positively correlated with emotion-focused coping strategy, active-avoidant, religious/denial, and also positively correlated with the subscales of perceived social support.

**Table 3:** Correlation among all the study variables (N = 352) AA: active avoidant; PF: problem-focused; R/D: religious/denial; EF: emotion-focused; FM: family; FS: friends; O: others; Psy. Tr: psycho-trauma; PSS: perceived social support; CS: coping strategy; **: p<0.01, *: p<0.05 considered significant

Variables	Psy. tr	PSS	FM	FS	O	CS	AA	PF	R/D	EF
Psy. tr	-	-0.01	0.02	-0.21^**^	-0.02	-0.19^**^	-0.10^*^	-0.18^**^	-0.29^**^	0.11^*^
PSS	-	-	0.85^**^	0.31^**^	0.84^**^	0.29^**^	0.42^**^	0.27^**^	0.11^*^	-0.14^**^
FM	-	-	-	0.31^**^	0.58^**^	0.30^**^	0.41^**^	0.27^**^	0.11^*^	0.17^**^
FS	-	-	-	-	0.25^**^	0.63^**^	0.57^**^	0.66^**^	0.48^**^	0.42^**^
O	-	-	-	-	-	0.25^**^	0.34^**^	0.24^**^	0.10^*^	0.11^*^
CS	-	-	-	-	-	-	0.87^**^	0.93^**^	0.66^**^	0.88^**^
AA	-	-	-	-	-	-	-	0.74^**^	0.39^**^	0.75^**^
PF	-	-	-	-	-	-	-	-	0.59^**^	0.76^**^
R/D	-	-	-	-	-	-	-	-	-	0.43^**^
EF	-	-	-	-	-	-	-	-	-	-

Table 4 shows that psycho-trauma explained 0.019 (19%) of the variance in predicting coping strategies. Perceived social support collectively explained 0.029 (29%) variance in predicting coping strategies, while its subscales, family, friends, and significant others, showed 0.03 (3%), 0.063 (63%), and 0.025 (25%), respectively.

**Table 4:** Simple linear regression predicting coping strategies from psycho-trauma and perceived social support (N = 352) S.E: standard error; Psy. tr: psycho-trauma; Sig others: significant others; LL:  lower limit; UL: upper limit; *p < 0.05, **p < 0.01 considered significant

Variables	B	S.E	β	LL (95%CI)	UL (95%CI)
Constant	98.21	7.31	-	83.84	112.58
Psy. tr.	-0.76	0.21	-0.19	-1.17	-0.35
R^2^	0.04	-	-	-	-
F	13.34***	-	-	-	-
Constant	51.25	3.6		44.16	58.34
PSS	0.43	0.07	0.29	0.28	0.58
R^2^	0.09	-	-	-	-
F	33.41***	-	-	-	-
Constant	53.04	3.2	-	46.73	59.34
Family	1.15	0.19	0.3	0.77	1.53
R^2^	0.09	-	-	-	-
F	35.52***	-	-	-	-
Constant	35.86	2.38	-	31.16	40.55
Friends	3.86	0.25	0.63	3.37	4.35
R^2^	0.4	-	-	-	-
F	238.99***	-	-	-	-
Constant	58.27	2.87	-	52.62	63.91
Sig. others	0.87	0.18	0.25	0.51	1.22
R^2^	0.06	-	-	-	-
F	23.25***	-	-	-	-

Table 5 shows that the combined effect of perceived social support and psycho-trauma was found to be significant, and it added a unique variance of 0.015 (15%) in explaining coping strategies. Overall, it was clear that perceived social support moderates the role of experience of psycho-trauma and coping strategies.

**Table 5:** Hierarchical regression analysis showing the effect of perceived social support in predicting coping strategy (N = 352) CI: confidence interval; LL: lower limit; UL: upper limit; ***p =0.00 considered significant

Variables	Β	R	P	LL	UL
Constant	184.21***	-	0.00	125.07	243.36
Perceived social support	-1.97***	-	0.00	-3.27	-0.68
Psycho-trauma	-3.82***	-	0.00	-5.51	-2.13
-	-	-	-	-	-
Interaction	-	0.07	0.00	0.03	0.11
R^2^	-	0.15	-	-	-
F	-	13.53***	-	-	-
ΔR^2^	-	0.03	-	-	-

**Figure 1:**
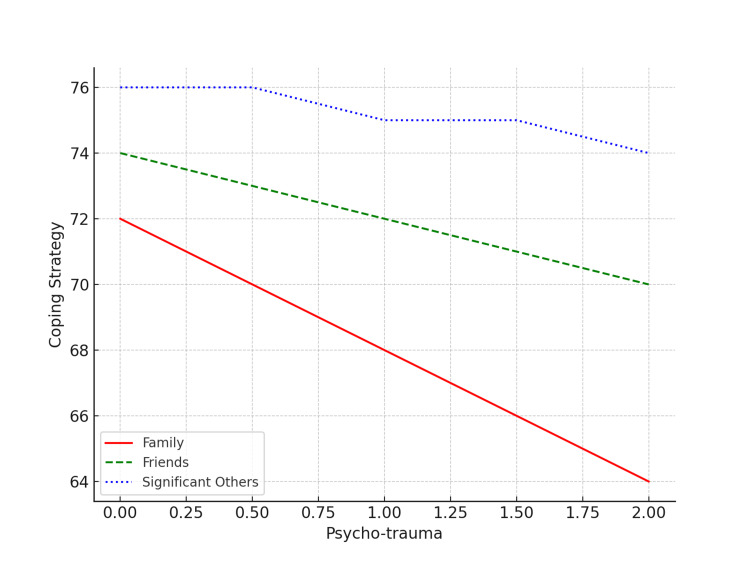
Moderating effect of perceived social support in the relationship between experience of psycho-trauma and coping strategies

Figure 1 shows that those individuals who had better coping strategies experienced less psychological trauma, and this effect was more obvious for those who were getting social support from their families. Almost similar results were found for the individuals who were getting social support from their friends. For them, better coping strategies resulted in experiencing less psychological trauma. No significant effect was found on psychological trauma and coping strategies for those who were getting support from significant others.

Table 6 shows the mean scores, standard deviations, and t-scores of men and women experiencing psycho-trauma, perceived social support, and coping strategies along with their subscales. Results indicated that men scored significantly higher than women on problem-focused coping, while women scored higher than men on emotion-focused coping. However, there were no significant differences between men and women in trauma experience.

**Table 6:** Differences in trauma experience among males and females M: mean, SD: standard deviation, LL: lower limit, UL: upper limit, AA: active avoidant; PF: problem-focused; R/D: religious/denial; EF: emotion-focused; FM: family; FS: friends; O: others; Psy.tr: psycho-trauma; PSS: perceived social support; CS: coaching strategy

Variables	Male		Female		T	P	95%CI		Cohen's d
-	(n =135)		(n = 217)		-	-	-		-
-	M	SD	M	SD	T	P	LL	UL	
Psy. Tr	34.41	3.81	35.45	3.41	2.67	0.05	-1.81	-0.28	0.29
PSS	46.92	8.8	46.27	10.08	0.60	0.00	-2.71	-1.43	0.06
FM	16.28	3.48	16.11	3.92	0.40	0.07	-0.64	0.98	0.04
FS	9.55	2.31	9.08	2.35	1.83	0.79	-0.03	0.97	0.2
O	15.33	3.81	15.36	4.23	0.05	0.00	-0.09	0.85	0.01
CS	75.11	11.29	69.51	15.41	3.65	0.00	2.58	8.61	0.41
AA	20.03	3.61	19.61	4.87	1.99	0.00	-0.54	1.37	0.09
PF	26.73	4.35	23.31	5.43	4.66	0.02	1.49	3.67	0.52
R/D	10.19	2.42	9.00	2.84	4.06	0.01	0.62	1.78	0.45
EF	17.18	3.19	18.58	4.56	3.12	0.00	0.52	2.28	0.35

Table 7 illustrates that there were significant differences in problem-focused strategy, a subscale of the PSS family, and BTQ. Students who experienced trauma multiple times scored high on emotion-focused coping. Students who did not experience trauma perceived support from family and scored high on this variable.

**Table 7:** One-way analysis of variance (ANOVA) for comparison of nature/kind of trauma experienced among young adults on study variables (N = 352) AA: active avoidant; PF: problem-focused; R/D: religious/denial; EF: emotion-focused; FM: family; FS: friends; O: others; BTQ: Brief Trauma Questionnaire; PS: perceived social support; BCI: Brief COPE Inventory

Variable	None		Once		Twice		Multiple		Values	
	(n = 158)		(n = 94)		(n = 43)		(n =57)			
-	M	SD	M	SD	M	SD	M	SD	F	P
Psy. Tr	36.16	3.54	34.97	3.07	32.86	3.14	33.77	3.75	14.15	0.00
PSS	47.7	10	45.28	9.37	47.26	10.12	45.67	8.2	1.52	0.20
FM	16.7	3.57	15.61	3.8	16.65	4.25	15.32	3.53	2.99	0.03
FS	9.46	2.69	8.76	1.96	9.4	2.18	9.44	1.88	1.99	0.11
O	15.47	4.22	14.98	3.86	15.49	4.03	15.51	4.04	0.35	0.78
CS	70.26	17.98	72.55	9.62	72.74	10.66	73.25	10.56	0.95	0.41
AA	19.49	5.37	19.97	3.32	19.35	4.09	20.54	3.19	0.97	0.40
PF	24.26	6.27	24.99	3.75	25.77	4.28	24.75	4.43	1.09	0.35
R/D	9.49	2.88	9.31	2.64	9.84	1.99	9.32	3.03	0.42	0.73
EF	17.02	5.26	18.29	2.76	17.79	3.18	18.63	2.56	3.07	0.02

Table 8 illustrates the mean variance of variables and shows that education has significant differences. The result also shows significant results for positive coping, and post hoc results demonstrate that students of MPhil showed higher scores than graduates. There was a significant difference in subscale friends of perceived social support, and students enrolled in masters received more support than graduates. Also, students of masters showed significant differences in psycho-trauma to the students of matriculation, graduate, and PhD.

**Table 8:** Difference of level of trauma among various education levels AA: active avoidant; PF: problem-focused; R/D: religious/denial; EF: emotion-focused; FM: family; FS: friends; O: others; Psy.tr: psycho-trauma; PSS: perceived social support; CS: coaching strategy; MPhil: Master of Philosophy; PhD: Doctor of Philosophy

Variable	Graduation		Masters		MPhil		PhD		Values	
	(n=150)		(n=159)		(n=40)		(n=3)			
-	M	SD	M	SD	M	SD	M	SD	F	P
Psy. Tr	34.59	3.53	35.59	3.58	34.9	3.7	34.9	3.7	2.98	0.03
PSS	45.63	9.71	47.73	9.43	46.8	9.58	40.67	10.01	1.64	0.17
FM	15.91	3.94	16.39	3.56	16.45	3.79	14.33	3.05	0.73	0.53
FS	8.93	1.97	9.43	2.57	9.9	2.44	7.67	2.88	2.76	0.04
O	14.86	4.35	15.92	3.68	14.95	4.29	14.67	3.51	1.95	0.12
CS	72.02	10.89	69.67	16.96	78.78	10.67	64	16.46	4.84	0.00
AA	19.9	3.68	19.42	5.19	21	3.44	16	3.6	2.15	0.02
PF	24.69	3.99	24.13	6.12	27.4	4.13	21.33	7.02	4.81	0.00
R/D	9.32	2.87	9.33	2.7	10.43	2.24	9.67	3.21	1.9	0.12
EF	18.11	2.87	16.79	5.03	19.95	3.13	17	4.35	7.37	0.00

Table 9 illustrates a one-way analysis of variance (ANOVA) to compute the mean differences of psycho-trauma, perceived social support, and coping strategies along with their subscales. This analysis produced significant results of psycho-trauma. The mean of fathers with MPhil education was high as compared to graduates and undergraduates, which showed their children experience more trauma as compared to undergraduates. The mean of fathers with PhD education scored higher on subscales of perceived social support significant others as compared to other study variables, and the mean of fathers with no education scored higher on problem-focused coping as compared to other variables. The mean of fathers with no education scored higher on religious/denial coping as compared to other study variables. Positive coping had a significant difference in the means of fathers with no education than other study variables.

**Table 9:** Comparison of paternal education on experiencing psycho-trauma, perceived social support, and coping strategy along with their subscales M: mean; SD: standard deviation; Psy.tr: psycho-trauma; PSS: perceived social support; FM: family; FS: friends; O: others; CS: coping strategy; AA: active avoidant; PF: problem-focused; R/D: religious/denial; EF: emotion-focused; MPhil: Master of Philosophy; PhD: Doctor of Philosophy

Scales	Primary		Matric		Graduate		MPhil		PhD		Others		Values	
	(N=8)		(N=93)		(N=74)		(N=135)		(N=36)		(N=6)			
-	M	SD	M	SD	M	SD	M	SD	M	SD	M	SD	F	P
Psy.tr	34.5	3.82	34.96	3.97	35.31	3.52	35.47	3.37	33.28	3.87	35.33	2.25	2.29	0.05
PSS	45.88	7.83	45.4	10.02	46.97	9.23	46.05	9.48	51.86	9.36	46.5	5.86	2.63	0.02
FM	15.63	4.31	15.99	3.86	16.04	3.39	15.89	3.89	18.03	3.26	17	3.35	2.1	0.06
FS	10.25	2.19	9.45	1.78	9.26	2.33	8.95	2.54	9.67	2.92	9.5	1.64	1.12	0.35
O	15.75	3.41	14.48	4.35	15.29	3.82	15.16	4.02	17.25	3.62	14	3.52	2.99	0.01
CS	79.38	14.56	75.65	8.71	72.65	11.25	69.7	17.67	64.94	14.14	71.67	7.7	4.32	0.00
AA	21	4.5	20.6	3.2	19.55	3.57	19.66	5.68	18.22	3.25	19.83	1.94	1.73	0.13
PF	28.38	6.07	26.07	3.43	25.09	4.41	23.73	6.26	23.17	5.07	25.83	2.23	3.98	0.00
R/D	10.63	2.97	9.9	2.34	10.01	2.64	8.76	3.05	9.69	2.34	8.17	1.33	3.56	0.00
EF	19.38	3.42	19.08	2.23	17.99	3	17.55	4.57	13.86	5.91	17.83	3.66	9.67	0.00

Table 10 shows significant differences in psycho-trauma and religious/denial coping. The mean of mothers with matriculation education was high as compared to graduates and PhDs, which showed their children experience more trauma. Subscale religious/denial coping had a significant difference in the means of mothers' education categories, which showed the children of uneducated mothers scored high on religious/denial coping.

**Table 10:** Differences based on maternal education about the study variables among young adults (N = 352) M: mean; SD: standard deviation; Psy.tr: psycho-trauma; PSS: perceived social support; FM: family; FS: friends; O: others; CS: coping strategy; AA: active avoidant; PF: problem focused; R/D: religious/denial; EF: emotion focused; MPhil: Master of Philosophy; PhD: Doctor of Philosophy

Variable	Primary (N =99)		Matric (N = 131)		Graduate (N = 79)		MPhil (N = 36)		PhD (N = 7)		Values	
Variable	M	SD	M	SD	M	SD	M	SD	M	SD	F	P
Psy. tr	34.26	3.85	36.04	3.16	34.85	3.33	34.17	4.12	34.57	4.31	4.46	0.00
PSS	47.18	10.32	45.79	9.27	47.06	10.02	47.31	7.46	48.14	11.71	0.46	0.77
FM	16.27	3.95	16.15	3.71	15.97	3.97	16.39	2.63	16.57	4.96	0.12	0.97
FS	9.73	2.23	9.20	2.57	9.03	1.89	8.94	2.50	8.00	2.71	1.89	0.11
O	15.56	4.04	14.76	4.21	15.82	3.99	16.00	3.43	14.86	5.30	1.29	0.27
CS	71.28	12.66	71.70	17.12	70.75	10.40	75.53	15.00	76.86	9.04	0.49	0.75
AA	19.26	3.81	19.66	5.31	20.30	3.48	20.06	4.52	21.71	3.35	1.01	0.40
PF	24.93	4.46	24.90	6.12	23.68	4.44	25.33	5.00	26.71	3.64	1.26	0.29
R/D	10.01	2.01	9.32	2.89	8.81	2.98	9.75	3.22	9.86	2.48	2.36	0.05
EF	17.07	4.89	17.82	4.36	17.95	2.50	18.39	4.28	18.57	1.40	1.00	0.41

## Discussion

The study aimed to explore the impact of psycho-trauma experience and social support on young adults' coping strategies while also examining demographic factors. The study found that psycho-trauma experiences are negatively associated with perceived social support and problem-focused coping strategies, while positively associated with emotion-focused coping strategies, and this is in line with previous research [19]. The experience of psycho-trauma can also decrease trust, impacting daily life functioning. Results also showed that this hypothesis was accepted (Table 3).

Findings showed that perceived social support is positively associated with problem-focused coping strategy and negatively related to emotion-focused coping strategy [20]. The study highlights the positive impact of social support on individuals' motivation and problem-solving abilities, while emotion-focused coping mechanisms such as projection and avoidance can negatively impact situations. Results related to the hypothesis mentioned just above were supported by the existing literature (Table 4).

Research indicates that perceived social support moderates the relationship between psycho-trauma experience and coping strategy, with strong predictors of psycho-trauma and covariates in regression models [21]. Results also showed that perceived social support moderates the relationship between the experience of psycho-trauma and coping strategy (Table 5).

Gender differences show women tend to have less social support and more emotion-focused coping, while men tend to have more problem-focused coping. These findings can be explained in the light of earlier work [22]. The study reveals that while there are differences between the genders in how they handle stress, there are more similarities in daily stress management, and these differences are more prevalent among men and women rather than across agencies.

Men show extra self-efficacy; this might be the result of near links to their activity [23]. It has been shown that men usually use problem-solving as a coping method. This sort of coping is generally related to men, as they often act realistically and rationally and pay less interest to emotional elements related to stressful conditions [24]. Women scored significantly higher than men in emotional and avoidance coping styles, even as scoring lower in rational and detachment coping styles [25]. 

The results of this study highlight the essential role of social backing in moderating the influence of psycho-trauma on coping approaches among young adults. This aligns with the combined bio-psychosocial model for post-traumatic stress recovery, which emphasizes the interaction between biological, psychological, and social factors in stress recovery [26].

Moreover, the study's results on gender differences in coping strategies provide essential insights. Females, experiencing higher rates of psycho-trauma, tend to use more emotion-focused coping strategies, such as avoidance and denial. This finding is consistent with previous research indicating that women are more likely to engage in emotional and avoidance coping mechanisms, which can exacerbate psychological distress [27]. On the other hand, males demonstrate higher use of problem-focused coping strategies, which are generally associated with better mental health outcomes.

Finally, the study underscores the urgent need for culturally compatible mental health interventions in Pakistan and Afghanistan, particularly in regions severely affected by ongoing violence, such as Swat and Nangarhar. Implementing evidence-based models of healthcare, as supported by previous research in similar contexts, could significantly enhance the mental health services available to trauma survivors in these areas [28,29]. Moreover, increasing awareness and training programs for mental health professionals could help address the current gaps in resources and policy implementation.

The study on coping strategies among young adults from just two cities has limitations due to its quantitative nature and the inclusion of severely ill participants. It is recommended to study qualitatively, considering actual responses to stressors. The quantitative nature may also limit responses to specific behaviors during coping, and the diplomatic attitude towards psychological illness may limit evaluations due to social expectations. More research is needed to increase awareness and understanding.

The study provides valuable insights for clinicians and mental health professionals on psychological distress, trauma, and their relationship with perceived social support and coping strategies among young adults, offering guidelines for further research, especially in Pakistan and Afghanistan, where there is limited information on these topics.

## Conclusions

The study suggests that a qualitative exploration of coping strategies and mechanisms could be beneficial, aiding in planning interventions and prevention for individuals who think negatively about life events. Reducing psycho-trauma can be achieved by adopting positive coping styles and reducing repetitive negative thoughts. The study emphasizes the importance of students having proper knowledge and skills to cope with daily challenges. Results show a significant positive relationship between perceived social support and coping strategy, while negatively related to psycho-trauma. The study was first conducted in two cities, Pakistan and Afghanistan, highlighting the need for further exploration in the relevant field. The findings help us understand health-related issues, future orientation, and factors like traumatic events affecting students' lives.

## Appendices

Appendix A 

**Table 11:** Brief Trauma Questionnaire The Brief Trauma Questionnaire (BTQ), derived from the Short Trauma Interview, was used to assess exposure to traumatic events based on DSM-5 Criterion A. The BTQ focuses on life-threatening or severely injurious experiences. Short Trauma Interview [15].

Event	Has this ever happened to you?	If the event happened, did you think your life was in danger or you might be seriously injured?	If the event happened, were you seriously injured?
1. Have you ever served in a war zone, or have you ever served in a noncombat job that exposed you to war-related casualties (for example, as a medic or on graves registration duty?)	No Yes	No Yes	No Yes
2. Have you ever been in a serious car accident, or a serious accident at work or somewhere else?	No Yes	No Yes	No Yes
3. Have you ever been in a major natural or technological disaster, such as a fire, tornado, hurricane, flood, earthquake, or chemical spill?	No Yes	No Yes	No Yes
4. Have you ever had a life-threatening illness such as cancer, a heart attack, leukemia, AIDS, multiple sclerosis, etc.?	No Yes	No Yes	NA
5. Before age 18, were you ever physically punished or beaten by a parent, caretaker, or teacher so that: you were very frightened, or you thought you would be injured; or you received bruises, cuts, welts, lumps, or other injuries?	No Yes	No Yes	No Yes
6. Not including any punishments or beatings you already reported in Question 5, have you ever been attacked, beaten, or mugged by anyone, including friends, family members, or strangers?	No Yes	No Yes	No Yes
7. Has anyone ever made or pressured you into having some type of unwanted sexual contact? Note: By sexual contact, we mean any contact between someone else and your private parts or between you and someone else’s private parts	No Yes	No Yes	No Yes
8. Have you ever been in any other situation in which you were seriously injured, or have you ever been in any other situation in which you feared you might be seriously injured or killed?	No Yes	NA	No Yes
9. Has a close family member or friend died violently, for example, in a serious car crash, mugging, or attack?	No Yes	NA	No Yes
10. Have you ever witnessed a situation in which someone was seriously injured or killed, or have you ever witnessed a situation in which you feared someone would be seriously injured or killed? Note: Do not answer “yes” for any event you already reported in Questions 1-9	No Yes	NA	NA

Appendix B 

**Table 12:** The Multidimensional Scale of Perceived Social Support (MSPSS), developed by Rafai Source: [16]

-	Very strongly disagree	Strongly disagree	Mildly disagree	Neutral	Mildly agree	Strongly agree	Very strongly agree
There is a special person who is around when I am in need	1	2	3	4	5	6	7
There is a special person with whom I can share joys and sorrows	1	2	3	4	5	6	7
My family really tries to help me	1	2	3	4	5	6	7
I get the emotional help & support I need from my family	1	2	3	4	5	6	7
I have a special person who is a real source of comfort to me	1	2	3	4	5	6	7
My friends really try to help me	1	2	3	4	5	6	7
I can count on my friends when things go wrong	1	2	3	4	5	6	7
I can talk about my problems with my family	1	2	3	4	5	6	7
I have friends with whom I can share my joys and sorrows.	1	2	3	4	5	6	7
There is a special person in my life who cares about my feelings	1	2	3	4	5	6	7
My family is willing to help me make decisions	1	2	3	4	5	6	7
I can talk about my problems with my friends	1	2	3	4	5	6	7

Appendix C 

**Table 13:** Brief Coping Orientation to Problems Experienced (COPE) Inventory Developed by Carver, this questionnaire was used to assess coping strategies [17].

Statement	I haven't been doing this at all	A little bit	A medium amount	I’ve been doing this a lot
I've been turning to work or other activities to take my mind off things.	1	2	3	4
I've been concentrating my efforts on doing something about the situation I'm in.	1	2	3	4
I've been saying to myself 'this isn't real'.	1	2	3	4
I've been using alcohol or other drugs to make myself feel better.	1	2	3	4
I've been getting emotional support from others.	1	2	3	4
I've been giving up trying to deal with it.	1	2	3	4
I've been taking action to try to make the situation better.	1	2	3	4
I've been refusing to believe that it has happened.	1	2	3	4
I've been saying things to let my unpleasant feelings escape.	1	2	3	4
I’ve been getting help and advice from other people.	1	2	3	4
I've been using alcohol or other drugs to help me get through it.	1	2	3	4
I've been trying to see it in a different light, to make it seem more positive.	1	2	3	4
I’ve been criticizing myself.	1	2	3	4
I've been trying to come up with a strategy about what to do.	1	2	3	4
I've been getting comfort and understanding from someone.	1	2	3	4
I've been giving up the attempt to cope.	1	2	3	4
I've been looking for something good in what is happening.	1	2	3	4
I've been making jokes about it.	1	2	3	4
I've been doing something to think about it less, such as going to movies, watching TV, reading, daydreaming, sleeping, or shopping.	1	2	3	4
I've been accepting the reality of the fact that it has happened.	1	2	3	4
I've been expressing my negative feelings.	1	2	3	4
I've been trying to find comfort in my religion or spiritual beliefs.	1	2	3	4
I’ve been trying to get advice or help from other people about what to do.	1	2	3	4
I've been learning to live with it.	1	2	3	4
I've been thinking hard about what steps to take.	1	2	3	4
I’ve been blaming myself for things that happened.	1	2	3	4
I've been praying or meditating.	1	2	3	4
I've been making fun of the situation.	1	2	3	4

## References

[1] Flannery RB (2022). Psychological trauma and the trauma surgeon. Psychiatr Q.

[2] (2024). Injuries and violence. https://www.who.int/news-room/fact-sheets/detail/injuries-and-violence.

[3] May CL, Wisco BE (2016). Defining trauma: how level of exposure and proximity affect risk for posttraumatic stress disorder. Psychol Trauma.

[4] Yehuda R, Hoge CW, McFarlane AC (2015). Post-traumatic stress disorder. Nat Rev Dis Primers.

[5] Center for Substance Abuse Treatment (US) (2014). Chapter 3, understanding the impact of trauma. Treatment Improvement Protocol (TIP) Series, No. 57.

[6] Williams P, Barclay L, Schmied V (2004). Defining social support in context: a necessary step in improving research, intervention, and practice. Qual Health Res.

[7] Wei W, Li X, Tu X, Zhao J, Zhao G (2016). Perceived social support, hopefulness, and emotional regulations as mediators of the relationship between enacted stigma and post-traumatic growth among children affected by parental HIV/AIDS in rural China. AIDS Care.

[8] Harandi TF, Taghinasab MM, Nayeri TD (2017). The correlation of social support with mental health: a meta-analysis. Electron Physician.

[9] Liu Z, Zhao X, Zhao L, Zhang L (2023). Relationship between perceived social support and mental health among Chinese college football athletes: a moderated mediation model. BMC Psychol.

[10] Algorani EB, Gupta V (2024). Coping Mechanisms. https://www.ncbi.nlm.nih.gov/books/NBK559031/.

[11] Tomsis Y, Gelkopf M, Yerushalmi H, Zipori Y (2018). Different coping strategies influence the development of PTSD among first-time mothers. J Matern Fetal Neonatal Med.

[12] Baqutayan SMS (2015). Stress and coping mechanisms: a historical overview. Mediterr J Soc Sci.

[13] The Lancet Global Health (2020). Mental health matters. Lancet Glob Health.

[14] Jibeen T (2016). Perceived social support and mental health problems among Pakistani young adults. Community Ment Health J.

[15] Schnurr P, Vielhauer M, Weathers F (1995). Brief Trauma Questionnaire (BTQ). https://www.ptsd.va.gov/professional/assessment/te-measures/brief_trauma_questionnaire_btq.asp#:~:text=The%20BTQ%20was%20originally%20designed,a%20brief%20self%2Dreport%20format..

[16] Zimet GD, Powell SS, Farley GK, Werkman S, Berkoff KA (1990). Psychometric characteristics of the multidimensional scale of perceived social support. J Pers Assess.

[17] Carver CS (1997). You want to measure coping but your protocol's too long: consider the brief COPE. Int J Behav Med.

[18] Akhtar M (2005). Coping strategies and their relationship with stress and time demand among university students.

[19] Zalta AK, Tirone V, Orlowska D (2021). Examining moderators of the relationship between social support and self-reported PTSD symptoms: a meta-analysis. Psychol Bull.

[20] Maheux A, Price M (2016). The indirect effect of social support on post-trauma psychopathology via self-compassion. Pers Individ Differ.

[21] Wang J, Mann F, Lloyd-Evans B, Ma R, Johnson S (2018). Associations between loneliness and perceived social support and outcomes of mental health problems: a systematic review. BMC Psychiatry.

[22] Liddon L, Kingerlee R, Barry JA (2018). Gender differences in preferences for psychological treatment, coping strategies, and triggers to help-seeking. Br J Clin Psychol.

[23] Freire C, Ferradás MD, Regueiro B, Rodríguez S, Valle A, Núñez JC (2020). Coping strategies and self-efficacy in university students: a person-centered approach. Front Psychol.

[24] Spendelow JS, Adam LA, Fairhurst BR (2017). Coping and adjustment in informal male carers: a systematic review of qualitative studies. Psychol Men Masc.

[25] Kersh R (2017). Women in higher education: exploring stressful workplace factors and coping strategies. NASPA J Women High Educ.

[26] Calhoun CD, Stone KJ, Cobb AR, Patterson MW, Danielson CK, Bendezú JJ (2022). The role of social support in coping with psychological trauma: an integrated biopsychosocial model for posttraumatic stress recovery. Psychiatr Q.

[27] Rathakrishnan B, Bikar Singh SS, Yahaya A (2022). Perceived social support, coping strategies, and psychological distress among university students during the COVID-19 pandemic: An exploration study for social sustainability in Sabah, Malaysia. Sustainability.

[28] Akbar Z, Aisyawati MS (2021). Coping strategy, social support, and psychological distress among university students in Jakarta, Indonesia during the COVID-19 pandemic. Front Psychol.

[29] Sharp P, Oliffe JL, Kealy D, Rice SM, Seidler ZE, Ogrodniczuk JS (2023). Social support buffers young men's resilient coping to psychological distress. Early Interv Psychiatry.

